# Surgery

**Published:** 1891-06

**Authors:** 


*Surgery*. By C. W. MANSELL MOULLIN, M.A., M.D., F.R.C.S. Pp. 1413. London: J. & A. CHURCHILL. 1891.

The issue of a new text-book on Surgery of the size and pretension of the work before us is in itself a noteworthy event. There is a demand for a good text-book on Surgery; for our old ones have, most of them, fallen into dubious, or even bad, repute, and we are ready to welcome a new one with a mind that is even better than unbiassed. We sat down to a study of the work in this hopeful mood, and we rose from its perusal congratulating its author on the fulfilment of his task.

The mere labour of writing a work of this size is no mean undertaking. In bulk it surpasses most of our English students' text-books, and is exceeded only by the single-author works of Gross and Agnew in America. In variety of subjects treated, in breadth and comprehensiveness, it probably surpasses all. Well as our author has done, we believe he would have done better had he called in more extraneous help: the excellent character of the four articles by four authors, which form a very small proportion of the bulk of the work, confirms us in this belief, which is further strengthened by the undoubted existence of certain weak elements in the work. Such weak points might have been removed if the sheets, as they passed through the press, had been submitted to the criticism of surgical friends specially qualified for the task. Even if such criticism had not changed opinions, it would certainly have done good by bringing forth fuller evidence in favour of dubious principles. As an example, we might instance the author's description of Colles' fracture of the radius. Without anything like sufficient proof adduced, the causation of this fracture is described in a manner directly antagonistic to the views of our most competent authorities. A critical friend would certainly have made our author re-write this description; he would have pointed out that if these were our author's views, they should, in the first place, have been put forth and demonstrated in an elaborate monograph. The settled views of capable men, put forth in many writings, cannot be upset by a page in a text-book. It is but fair to say that only in rare instances can the author's views be regarded as against the weight of authority. We believe that with such critical assistance as we have suggested, these few instances might be removed: perhaps another edition will see them removed.

In respect of the manner in which the book is written up to date, we must give unqualified and unstinted praise. In all the newest surgery of asepticism, in the most recent advances in the surgery of the intestines and the bladder and the brain, the writing is up almost to the day of publication. The most recent investigations and the latest surgical innovations scattered throughout journals are collected, elaborated, systematised, and made to do duty in their proper sequences throughout the work. Nothing, in our study of the work, has impressed us more favourably than this proof of the laborious energy of the author.

For some mysterious reason, which we have never been able to understand, medical works are arranged into chapters, like parcels according to bulk, and sometimes according to bulk only. In the case of a novel appearing in weekly numbers, the length of a chapter may have more than one meaning; it may even with propriety stop in the midst of a thrilling narrative; but such an arrangement is difficult to understand in a scientific work. For instance, Chapter XIV. is on "Injuries and Diseases of the Neck and Throat," and there we might naturally expect to find some account of Diseases of the Thyroid, and possibly of the Larynx; but it is not so. Diseases of the Thyroid have an independent chapter all to themselves, while Diseases of the Larynx are lumped up in Chapter XIII. with Diseases of the Ear! Again, Injuries and Diseases of the Abdomen are minutely and exhaustively described, down to surgical affections of the Liver and the Pancreas, in Chapter XVIII.; but the Kidney is rigidly excluded from this chapter, and appears in a separate chapter after one on Diseases of the Rectum. Even if there are sound reasons why the kidney should be excluded from the abdomen, or the prostate (Chapter XXII.) from the bladder (Chapter XXI.), which we doubt, we venture to affirm that it is certainly not proper to keep the urethra (Chapter XXIII.) out of the penis (Chapter XXIV.). This forced arrangement into chapters is not peculiar to our author by any means; it is probably less conspicuous in his case than in other text-books that could be named. Nevertheless, we regard it as culpable, and condemn it.

In minor details the classification is not always perfect.

For instance, Inflammation of Bone is described under the following heads:

1. Acute Inflammation.
2. Chronic Inflammation—Rarefying Ostitis, with the sub-heads of:
   (*a*) Resolution;
   (*b*) Persistence (Caries Sicca or Fungosa);
   (*c*) Organisation (Osteo-Sclerosis).
3. Caries:
   (*a*) Simple;
   (*b*) Specific.

Caries, it will be noted, is not a variety of chronic inflammation, it is not even a "rarefying ostitis." Osteo-Sclerosis is, however, a variety of "rarefying ostitis"! Then comes a section on Varieties of Inflammation, starting with *simple traumatic* (in italics), and going on with PHOSPHORUS NECROSIS (in capitals). Surely there are mistakes in type-setting here, as well as serious faults in actual classification!

As to the illustrations, our criticisms have, in a measure, been discounted. It is the wholesome practice of writers of books to acknowledge in the most prominent manner in the text, or with the description of the figure, the source from which an engraving, not original, has been derived. Our author has ignored this practice, demanded no less by honesty than by courtesy, until a grave complaint has been made by one most aggrieved. By issuing a new "List of Illustrations," giving the source of each borrowed engraving, Mr. Moullin did what he could to minimise a fault which nothing less than separate acknowledgments printed with each figure in the text can completely remove.

In conclusion, we can honestly speak of the work as being comprehensive in matter, clear in diction, trustworthy (with few exceptions) in fact, and on the whole worthy of being recommended as a text-book.

